# X-linked Adrenoleukodystrophy in a 20-Year-Old Male With an ABCD1 Gene Mutation: First Case From Pakistan

**DOI:** 10.7759/cureus.21837

**Published:** 2022-02-02

**Authors:** Mariam Ghori, Rameen A Molani, Prof Mohsina N Ibrahim, Misbah I Hanif, Jamal Jamal Raza

**Affiliations:** 1 Medicine, Jinnah Sindh Medical University, Karachi, PAK; 2 Pediatrics and Endocrinology, National Institute of Child Health, Karachi, PAK

**Keywords:** neuro-endocrine disorder, genetic panel test, genetic mutations, lorenzo’s oil, stem cell transplant, abcd1 gene, addison’s disease, x- linked adrenoleukodystrophy

## Abstract

X-linked adrenoleukodystrophy (X-ALD) is a rare neurodegenerative disease characterized by genetic mutation of the ABCD1 gene. This gene encodes for transmembrane adrenoleukodystrophy protein (ALDP). Defective ALDP protein results in the accumulation of a very long chain fatty acid (VLCFA) within certain tissues and plasma. X-ALD can initially present as Addison's disease (primary adrenal insufficiency) as the accumulation of VLCFA most importantly occurs in the adrenal gland. Our 20-year-old male patient, a known case of Addison's disease, presented with vision loss, neurologic symptoms, and psychiatric issues. Neurologic symptoms included poor concentration and memory, while psychiatric problems included primarily depressive disorder and mild psychotic behavior.

His Addison's disease was secondary to X-ALD. Still, he was diagnosed late due to a lack of awareness of X-ALD and a lack of resources for genetic testing in Pakistan. Therefore, the purpose of this case report is to spread knowledge and understanding of X-ALD, so that it can be ruled out as the potential cause of adrenal insufficiency in young patients, particularly males diagnosed with Addison's disease. Moreover, if the patient presents with Addison's disease and psychiatric issues, they should be tested to rule out X-ALD.

## Introduction

Adrenoleukodystrophy (ALD) is an X-linked progressive neurodegenerative disorder. It is characterized by the accumulation of very long chain fatty acid (VLCFA) due to the mutation of ABCD1 gene-encoded protein adrenoleukodystrophy protein (ALDP) [1].

It has an incidence of 1:17,000 in males, majorly involving a mutation of the ABCD1 gene, making it the most common inherited peroxisomal disorder. Clinically, the phenotypes of X-linked adrenoleukodystrophy (X-ALD) in males range from Addison-only to cerebral ALD (CALD) involving three different stages: childhood, adolescence, and adult to adrenomyeloneuropathy (AMN). Addison-only X-ALD is characterized by adrenal insufficiency. In contrast, cerebral ALD is rapidly progressive myelopathy. Moreover, patients with X-ALD are slowly progressive and eventually develop AMN in adulthood at the third or fourth decade of life [2].

Clinical progression of this disease involves intellectual deficits, communication disorders, and eventually paralysis, coma, and death [1].

While the most common form of X-ALD involves the ABCD1 gene, the etiology of this disease is still unclear. Due to this, there are no effective treatment modalities and known cures in the late stages. However, early diagnosis and stem cell therapy may show significant results [3].

## Case presentation

A 20-year-old male, a resident of Karachi, Pakistan, with a known history of Addison's disease presented in the outpatient department of a tertiary care hospital with complaints of severe blurred vision, headache, dizziness, mood disturbances, poor concentration, leg cramps, sleep disturbances, and urinary frequency for a year. He had no family history of Addison's disease. He was born through a normal pregnancy and delivery. His birth weight and head circumference were normal as well. Parents were in a non-consanguineous marriage. His two elder siblings (one male and one female) had no related symptoms.

Addison's disease was diagnosed at the age of 13 years when he presented with hyperpigmentation of the skin, gums, lips, and mucous membranes. Other symptoms included fatigue, muscle weakness, weight loss, loss of appetite, low mood, and increased thirst. Tests revealed raised adrenocorticotropic hormone (ACTH) levels (>2000 pg/ml, the normal range of ACTH is 7.2-63.3 pg/ml) and low cortisol levels (04 μg/dl, the normal range of cortisol is 6-20 μg/dl). However, his complete blood count (CBC), serum electrolytes, liver function tests (LFTs), plasma renin, serum aldosterone, thyroid-stimulating hormone (TSH), hemoglobin A1c (Hba1c), chest X-ray, ultrasound of the upper abdomen, MRI of brain and pituitary fossa with contrast were within normal ranges. These labs were done to rule out other differentials of his symptoms. He has been on replacement therapy of hydrocortisone since then. He did not receive mineralocorticoid replacement therapy due to normal electrolyte levels. Moreover, his ACTH levels kept fluctuating over the months but normalized upon dose adjustment of hydrocortisone.

In addition, at the age of 17 years, he was diagnosed with major depressive disorder, obsessive features, and borderline psychiatric traits. Since then, he had been taking antipsychotic and antidepressant medications, including risperidone, olanzapine, and fluoxetine capsules.

During his recent visit, his physical examination, height, and weight were normal. In addition, his speech, intelligence, attention, gait, muscle tone, tendon reflexes were normal too. Moreover, the fundoscopic examination was normal. No abnormal hyperpigmentation of skin or mucous membranes was seen.

His investigations revealed raised ACTH (1300 pg/ml), low vitamin B12 (210 pg/ml, the normal is between 220 and 925 pg/ml), and normal vitamin D levels.

**Figure 1:**
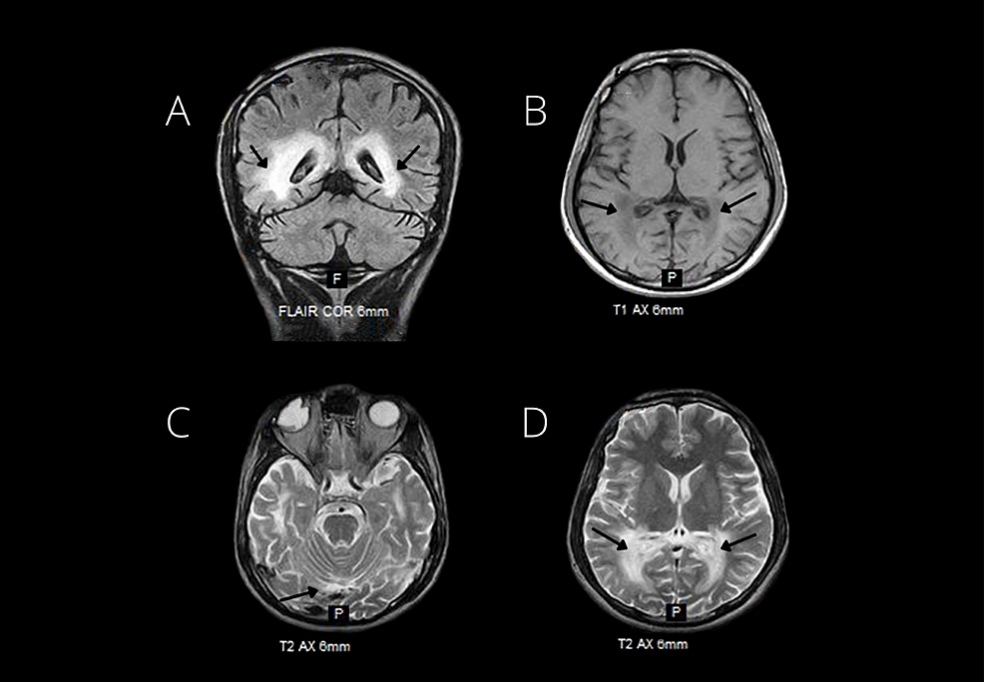
MRI brain scan of a 20-year-old male with a history of Addison's disease at the time of suspected X-ALD This shows bilateral symmetrical confluent areas of signal abnormalities in parieto-occipital, temporal lobe, and splenium of the corpus callosum. (A) Fluid-attenuated inversion recovery (FLAIR), showing hyperintensity or high signals in marked areas. (B) T1-weighted image shows hypointensity or low signals in the marked areas. (C) and (D) T2-weighted images also show hyperintensity or high signals in the marked areas. X-ALD - X-linked adrenoleukodystrophy

His MRI brain findings showed bilateral symmetrical confluent areas of signal abnormality involving parieto-occipital, temporal lobe, and splenium of the corpus callosum. This exhibited low signals on T1-weighted, high on T2-weighted, and fluid-attenuated inversion recovery (FLAIR) images shown in Figure 1.

In addition, visual evoked potential (VEP) interpretation showed absolute latencies of waves N-75, P-100, and N-145 were prolonged bilaterally with flash stimulation technique. His VEP was abnormal and showed bilateral optic pathway dysfunction. In contrast, the pattern reversal technique was not done due to poor patient compliance. His lipid profile showed raised triglycerides (169 mg/dl while normal is <150mg/dl), low density lipoprotein (LDL) (140mg/dl, the normal is <100 mg/dl), very low density lipoprotein (VLDL) (33.8 mg/dl, the normal is <30 mg/dl) , and low high density lipoprotein (HDL) (33 mg/dl, the normal is >40 mg/dl). Cholesterol levels were normal. Furthermore, his serum glutamic pyruvic transaminase (SGPT), creatinine levels, CBC, sodium, potassium, calcium levels, TSH, and blood sugar were normal.

**Figure 2:**
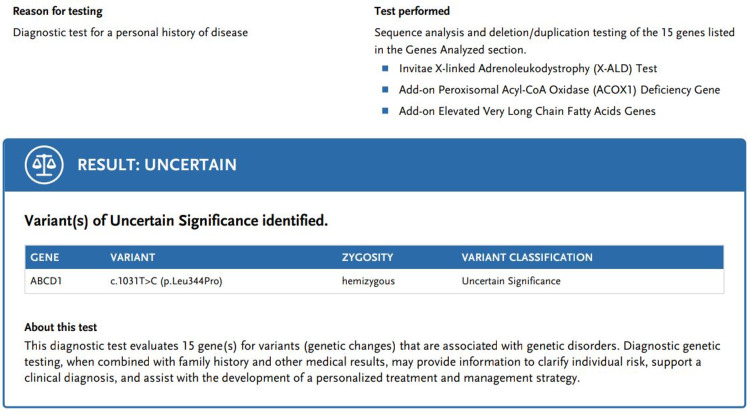
Genetic panel testing showing ABCD1 gene with variant c.1031T>C (p.Leu344Pro) and hemizygous zygosity

A genetic panel was ordered due to suspicion of X-ALD showing variants of uncertain significance (VUS) for the ABCD1 gene. It included the variant c.1031T>C (p.Leu344Pro) and hemizygous zygosity, shown in Figure 2. This change shows the replacement of leucine with proline at codon 344 of the ABCD1 protein.

Further, this change is not present in the population databases. We strongly suspected X-linked recessive adrenoleukodystrophy based on the genetic panel test, MRI brain findings, and clinical presentation. However, a very long chain fatty acid (VLCFA) test was not done due to the unavailability of the test in Pakistan.

So, he was prescribed Lorenzo's oil to prevent the further progression of the disease. He was taking a daily dose of Lorenzo's oil (5 ml), hydrocortisone (10 mg) twice a day, multi-vitamins, and antipsychotic drugs. He was also taking 1000 CC of vitamin B12 intramuscularly, once a month. Follow-up details included no changes in the condition of the patient.

## Discussion

In Pakistan, neurodegenerative diseases of genetic origin are not diagnosed and documented timely due to inadequate resources for genetic testing. However, they do contribute to a significant problem in the pediatric population. ALD is an X-linked progressive neurodegenerative disease most commonly involving the ABCD1 gene [1]. Mutation of the ABCD1 gene results in defective ALDP, a peroxisomal protein responsible for transporting VLCFA across transmembrane. Thus, causing accumulation of VLCFA within the tissues [4]. Therefore, we should conduct genetic panel testing and a VLFCA level test for early diagnosis.

X-ALD is a major cause of Addison's disease in boys. Addison's disease clinically presents with hyperpigmentation, often seen in ALD [3]. However, there is no direct correlation between ALD and hyperpigmentation. Moreover, ACTH levels higher than the normal limit (>100pg/ ml) help establish the diagnosis of Addison's disease. In our patient, proper cortisol replacement therapy (hydrocortisone) was initiated, due to which his cortisol and ACTH levels normalized. Fludrocortisone was not prescribed due to normal electrolyte levels. He also showed no hyperpigmentation.

Additional presentation involves visual defects, including visual field defects, loss of visual acuity, homonymous hemianopia, and cortical blindness [5]. In our patient, the ophthalmic assessment showed normal visual acuity. However, VEP suggested bilateral optic pathway dysfunction. Moreover, vision problems usually occur a few months after the appearance of neurological symptoms due to degenerative changes within the brain [5].

Diagnosis of ALD can be made by history, VLCFA levels, along with CT and MRI findings. In our patient, an MRI brain scan showed signal abnormalities in the temporal, parieto-occipital, and splenium of the corpus callosum due to bilateral and symmetrical confluent areas in the brain.

Usually, psychiatric issues develop before the deterioration of visual, auditory, and motor functions [6]. Our patient was diagnosed with major depressive disorder, obsessive features, and borderline psychiatric traits three years before presenting neurological symptoms.

Early screening and close observance of symptoms of adrenoleukodystrophy in children with Addison's disease should be considered for early diagnosis and treatment modalities. The treatment options include a hematopoietic stem cell transplant to seize the progression of demyelination of brain components in X-ALD [3].

In addition, Lorenzo's oil is used prophylactically in asymptomatic patients. It is suggested to normalize the levels of VLCFA (C24:0 and C26:0) in plasma levels of X-ALD. However, no significant effects on the progression are seen in patients with pre-existing neurological symptoms [7].

## Conclusions

X-linked Adrenoleukodystrophy is a rare neurodegenerative disorder that commonly presents as Addison's disease. Therefore, patients with Addison's disease should be screened for mutation of the ABCD1 gene using a genetic panel test. This will help in early diagnosis and timely utilization of the most effective therapy, hematopoietic stem cell therapy. Moreover, in countries where the confirmatory test for X-ALD, testing of VLCFA levels, is not available, the clinical presentation and genetic panel test can be focused on instead. 
